# Dedication of the Barlow Library of Los Angeles

**Published:** 1907-05

**Authors:** 


### DEDICATION OF THE BARLOW LIBRARY OF LOS ANGELES.

---

The establishment of medical libraries is so desirable a need of the medical profession and at the same time so unusual an event, that every such occasion seems worthy of special mention and record. It is, therefore, with considerable pride that Los Angeles, now a city of 260,000 persons, and the metropolis and natural geographical, financial, social and literary center of that wonderful region known as "The Great South-west," is able to announce to the world that the medical profession of that section has come into possession of a fire-proof medical library building and equipment costing more than thirty thousand dollars and that the institution starts its career of active work with more than five thousand volumes and publications.

For the generous gift of the building and equipment the medical profession is indebted to one of its own members, in fact, to one of the younger members of the profession of Los Angeles—Dr. W. Jarvis Barlow, who occupies the chair of clinical medicine in the College of Medicine of the University of Southern California. Dr. Barlow gave the building without any restrictions, but in his honor it has been called the Barlow Medical Library by the board of trustees, who were elected by the members. The books with which the institution starts constituted the former library of the College of Medicine of the University of Southern California. The control of the institution is in the hands of the patron members, these being physicians who subscribe twenty-five dollars annually. As yet there is no endowment and the salary of the librarian and the expense of publications come from dues of patron members, annual members (ten dollars yearly) and of associate members (five dollars yearly). —Geo. Kress, Jour. Am. A.

